# Distribution of Glycan Motifs at the Surface of Midgut Cells in the Cotton Leafworm (*Spodoptera littoralis*) Demonstrated by Lectin Binding

**DOI:** 10.3389/fphys.2017.01020

**Published:** 2017-12-08

**Authors:** Tomasz Walski, Kristof De Schutter, Kaat Cappelle, Els J. M. Van Damme, Guy Smagghe

**Affiliations:** ^1^Department of Crop Protection, Ghent University, Ghent, Belgium; ^2^Department of Molecular Biotechnology, Ghent University, Ghent, Belgium

**Keywords:** lectin, microvilli, glycosylation, *Spodoptera littoralis*, insect midgut, primary cell culture, cell polarization

## Abstract

Glycans are involved in many biological phenomena, including signal transduction, cell adhesion, immune response or differentiation. Although a few papers have reported on the role of glycans in the development and proper functioning of the insect midgut, no data are available regarding the localization of the glycan structures on the surface of the cells in the gut of insects. In this paper, we analyzed the spatial distribution of glycans present on the surface of the midgut cells in larvae of the cotton leafworm *Spodoptera littoralis*, an important agricultural pest insect worldwide. For this purpose, we established primary midgut cell cultures, probed these individual cells that are freely suspended in liquid medium with a selection of seven fluorescently labeled lectins covering a range of different carbohydrate binding specificities [mannose oligomers (GNA and HHA), GalNAc/Gal (RSA and SSA), GlcNAc (WGA and Nictaba) and Neu5Ac(α-2,6)Gal/GalNAc (SNA-I)], and visualized the interaction of these lectins with the different zones of the midgut cells using confocal microscopy. Our analysis focused on the typical differentiated columnar cells with a microvillar brush border at their apical side, which are dominantly present in the Lepidopteran midgut and function in food digestion and absorption, and as well as on the undifferentiated stem cells that are important for midgut development and repair. Confocal microscopy analyses showed that the GalNAc/Gal-binding lectins SSA and RSA and the terminal GlcNAc-recognizing WGA bound preferentially to the apical microvillar zone of the differentiated columnar cells as compared to the basolateral pole. The reverse result was observed for the mannose-binding lectins GNA and HHA, as well as Nictaba that binds preferentially to GlcNAc oligomers. Furthermore, differences in lectin binding to the basal and lateral zones of the cell membranes of the columnar cells were apparent. In the midgut stem cells, GNA and Nictaba bound more strongly to the membrane of these undifferentiated cells compared to the microvillar pole of the columnar cells, while SSA, HHA, WGA, and SNA-I showed stronger binding to the microvilli. Our results indicated that polarization of the midgut cells is also reflected by a specific distribution of glycans, especially between the basal and microvillar pole. The data are discussed in relation to the functioning and development of the insect midgut.

## Introduction

A growing amount of data underlines the biological importance of glycans at different levels of physiology of living organisms. Glycans found in cells are mostly linked to proteins via asparagine, serine/threonine or tryptophan as part of *N*-, *O*- or *C*-glycoproteins. Additionally, proteins can also be linked via serine or threonine to elaborated glycosaminoglycan chains (GAGs). All these glycans are built of a common set of monosaccharides, including, but not limited to mannose (Man), glucose (Glc), fucose (Fuc), galactose (Gal), xylose (Xyl), N-acetylglucosamine (GlcNAc), N-acetylgalactosamine (GalNAc), and sialic acid (Varki et al., 2009).

As in other organisms, glycans in insects are involved in a multitude of biological phenomena (reviewed in Walski et al., 2017). For instance, glycolipids take part in the epidermal growth factor signaling pathway, oocyte maturation and the formation of neuromuscular junctions (Chen et al., 2007; Pizette et al., 2009). *N*-glycosylation is important for insect immune responses (Herrero et al., 2007; Mortimer et al., 2012), nervous system functioning, movement and determines lifespan (Sarkar et al., 2006; Repnikova et al., 2010; Kato and Tiemeyer, 2013). Examples of *O*-linked glycan functions include mediation of cell adhesion and tracheal tube formation (Tian and Ten Hagen, 2007a,b; Zhang and Ten Hagen, 2011; Tran et al., 2012). Furthermore, *N*-linked glycosylation is essential for protein folding, stability, localization and function. For example, G-protein coupled receptors such as rhodopsin and dopamine receptors are known to be *N*-glycosylated (Schwarz and Aebi, 2011; Verlinden et al., 2014), and the presence of an appropriate glycan chain on *Drosophila melanogaster* rhodopsin is crucial for proper localization and photosensitivity of these proteins (Kunduri et al., 2014).

Furthermore, glycans play multiple roles in the insect digestive system. For instance, *O*-GalNAc is required for the development of the gut and its proper acidification (Tran and Ten Hagen, 2013) and maintaining the structure of the peritrophic matrix (Hegedus et al., 2009; Toprak et al., 2010a,b). Moreover, GalNAc residues decorating aminopeptidases and alkaline phosphatases are involved in Cry1Ac toxin binding (Angelucci et al., 2008; Rodrigo-Simón et al., 2008; Ning et al., 2010). In fact, most of the glycan epitopes present on the surface of the gut epithelium are possible binding targets for entomotoxic lectins and this protein-carbohydrate interaction is shown to be required to mediate their cytotoxic effects (Fitches et al., 2001, 2008; Cristofoletti et al., 2006; Hamshou et al., 2012; Walski et al., 2014).

In contrast to multiple examples of glycan significance for insect physiology, relatively little data is available with respect to glycan composition. The few available analyses focused either on whole organisms (Aoki et al., 2007) or single proteins (Kim et al., 2003; Knight et al., 2004) and rarely on specific tissues or structures, such as the digestive tract (Vancova et al., 2012). Clearly, more glycomic research will be of benefit to the basic knowledge of insect physiology and cell differentiation.

The glycan composition is most commonly analyzed using various mass spectrometry and chromatography techniques. These methods offer high sensitivity and reliability, but each type of glycan (*N*- or *O*-linked, GAGs, glycolipids) requires specific protocols and separate analysis (Gutternigg et al., 2007; Johswich et al., 2009; Mechref et al., 2009). Lectin microarrays can address this issue and are suitable for simultaneous analysis of all types of glycans, also on whole cells (Krishnamoorthy and Mahal, 2009; Rakus and Mahal, 2011). However, none of these techniques allows for analysis of spatial heterogeneity in glycan distribution on the surface of tissues or cells. Sensitive spatial analysis of different glycans is possible with MALDI imaging, yet this technology is still limited by the imaging resolution which is not high enough to analyze glycan topology at single cell level (Chaurand et al., 2007; Römpp and Spengler, 2013; Anderson et al., 2014). Therefore, to address the issue of concurrent analysis of different glycan types and their spatial distribution, microscopic techniques still remain an approach of choice. The use of metabolic glycan labeling (Ning et al., 2008), antibodies (Laughlin and Bertozzi, 2009) or lectins (Tian and Ten Hagen, 2007a; Mun et al., 2012) allows to analyze the occurrence of specific monosaccharides or glycan motifs across *N*-, *O*-glycans, glycolipids or GAGs simultaneously and precisely localize them taking advantage of the high resolution of electron or light microscopy.

The question of localization of different glycan epitopes on the cell surface is especially interesting for the insect gut. *In vivo*, the larval midgut of Lepidoptera consists of a pseudostratified epithelium of columnar and goblet cells, with stem cells located among their basolateral surfaces and resting on the basal lamina. The midgut columnar cells, also called enterocytes, are the most abundant cell type and typically are polarized into apical and basolateral poles. On the apical pole, these cells produce microvilli which are responsible for the secretion of enzymes and the absorption of nutrients (Lehane and Billingsley, 1996; Hakim et al., 2010). In the past, primary cultures of different insects have been prepared for studies on insect development, virus replication and midgut development and repair (Hakim et al., 2001; Loeb et al., 2003). In addition, effects of growth factors (Hakim et al., 2009; Loeb, 2010), hormones as ecdysone and juvenile hormone (Smagghe et al., 2006) and insecticidal toxins as *Bacillus thuringiensis* (Ning et al., 2010) have been investigated in these cultures. However, to our knowledge, these individual cells that occur freely in suspension in the liquid medium, have not been studied for the glycan motifs at their surface.

In this paper, we investigated the distribution of glycan motifs at the surface of two types of cells from the midgut epithelium of the cotton leafworm *Spodoptera littoralis*, that is a notorious agricultural pest insect damaging more than 40 plant species worldwide. After dissection of the larval midgut, primary cell cultures were established for the differentiated columnar cells that are dominantly present in the insect midgut, show a microvillar brush border zone at the apical side and function in food digestion and absorption, as well as for the undifferentiated stem cells that are important for midgut development and repair. Subsequently, we probed these individual cells with an array of seven fluorescently labeled lectins that cover a range of different carbohydrate binding specificities: mannose oligomers (GNA and HHA), GalNAc/Gal (RSA and SSA), GlcNAc (WGA and Nictaba) and Neu5Ac(α-2,6)Gal/GalNAc (SNA-I). Using confocal microscopy, lectin binding to the various cell surface regions (e.g., apical, basal, lateral) of these individual cells in the culture medium was measured. These primary cultures of individual cells allowed for the first time to investigate two hypotheses dealing with the importance of the glycan motifs for the insect midgut. First, we hypothesize that the apical/basolateral polarization of the differentiated columnar cells is reflected by a different distribution of glycans motifs, and if so this may determine the functionality of the midgut cells. Second, the glycosylation pattern of the stem cells may change during the differentiation process as reflected with a change in lectin binding. A difference in the lectin binding between differentiated columnar cells and undifferentiated stem cells, this would indicate that the differentiation of the cells during midgut development is associated with polarization and that this polarization is associated with differences in glycan distribution.

## Materials and methods

### Insects

A continuous colony of the cotton leafworm *S. littoralis* was maintained on an agar-based artificial diet under standardized conditions of 23–25°C, 60–70% relative humidity and a 16:8 (light:dark) photoperiod (Iga and Smagghe, 2011).

### Purification of lectins and labeling with FITC

GNA (*Galanthus nivalis* agglutinin) was isolated and purified from *G. nivalis* bulbs, HHA (*Hippeastrum* hybrid agglutinin) from *Hippeastrum* hybrid bulbs, WGA (wheat germ agglutinin) from *Triticum aestivum* germs, Nictaba from jasmonate-treated *Nicotiana tabacum* leaves, RSA (*Rhizoctonia solani* agglutinin) from the sclerotes of the fungus *R. solani*, SSA (*Sclerotinia sclerotiorum* agglutinin) from sclerotes of the fungus *S. sclerotiorum*, and SNA-I from lyophilized *Sambucus nigra* bark, with use of established protocols as previously described in Van Damme et al. (1988, 1996, 1998); Vandenborre et al. (2009) and Hamshou et al. (2010a, 2012). The purity of all lectins was confirmed by SDS-PAGE.

Lectins were labeled with fluorescein isothiocyanate (FITC) as described previously (Hamshou et al., 2010b). Briefly, lectins were dissolved in 50 mM sodium borate buffer (pH 8.5) and mixed with 24 fold molar excess FITC dissolved in dimethylformamide. After incubation at room temperature in the dark for 2 h, the free label was removed by gel filtration on a Sephadex G25 column equilibrated with PBS. Lectin activities in the eluted fractions were checked using agglutination assays (Van Damme et al., 1988) and the protein concentrations were determined with Bradford's method (Bradford, 1976). Since the ratio of moles of FITC to moles of lectin in the conjugate will vary between the different lectins, direct comparison between different lectins is not possible.

### Primary cell cultures from the midgut of *S. littoralis* larvae

Primary cultures of midgut cells were prepared from actively feeding last instars of *S. littoralis*. Dissected midguts were obtained as described in Cermenati et al. (2007) and cells dissociated for 1.5 h with 2 mg/ml of collagenase (Type I-AS; Sigma, Bornem, Belgium) in Insect Physiological Solution (IPS) (Cermenati et al., 2007). IPS mimics the osmolarity, pH and salt composition present in the hemolymph of Lepidopteran larvae. Collagenase treatment produced a culture constituted of a large number of columnar cells (the more abundant cell type in the culture, as *in vivo*), and a smaller number of goblet cells and stem cells.

### Evaluation of lectin interaction with columnar and stem cells of the larval midgut

Midgut columnar and stem cells were incubated for 1 h with 0.85 μM of FITC-labeled lectins. At this lectin concentration and using similar conditions of cell culture, the lectins do not to influence cell viability during the timeframe of the experiment, as shown previously (Shahidi-Noghabi et al., 2008; Vandenborre et al., 2008; Hamshou et al., 2010a,b; Caccia et al., 2012). Control cells were incubated with equal amounts of PBS. After incubation, the cells were fixed for 15 min with 4% paraformaldehyde in PBS. After three rinses with PBS, samples were mounted in Vectashield Mounting Medium (Vector Laboratories, Burlingame, CA).

The samples were examined under a confocal laser scanning microscope (Nikon A1R; Nikon Instruments, Paris, France), using a 60x/1.4 oil Plan Apo objective. A 488 nm laser was used for excitation of FITC-labeled lectins and for transillumination, and FITC fluorescence was detected through a 525/50 nm bandpass filter. Images were sampled at a pixel size of 83 × 83 nm and an optical section thickness of 1 μm. Using ImageJ software (http://imagej.nih.gov/ij/), mean pixel intensities were measured in the manually selected microvillar zone, basal, and lateral part of the membrane of columnar cells as well as in the cell membrane (perimeter) of stem cells. Fluorescence intensity, as a measure for the relative amount of lectin bound, was calculated separately for every imaged cell as a ratio of mean pixel intensity in a given zone over the background. After manual selection of the outline of the cell membrane, the fluorescence intensities were then normalized by calculating average pixel intensity within a zone. This normalization compensates for differences in membrane surface between microvillar, basal and lateral zone and allows semi-quantifying the amount of lectin binding over the cell membrane zones. The binding of each individual lectin between zones and cell types was analyzed and compared using *t*-tests in SPSS 22 Statistics 22 (IBM), *p*-values below 0.05 were chosen to indicate statistically significant differences.

### Verification of carbohydrate-binding specificity of lectins

FITC-labeled lectins (HHA, RSA and SNA-I) were pre-incubated for 30 min with a specific competing carbohydrate: 2 mg/ml of yeast mannans (Sigma) for HHA, 20 mM of GalNAc (Sigma) for RSA, 20 mM of α2,6-sialyllactosamine (Carbosynth) for SNA-I, or buffer for positive control. In addition, FITC-labeled RSA was pre-incubated for 30 min with 100 mM of the specific carbohydrate, GalNAc, or a non-specific carbohydrate, such as GlcNAc (Sigma). Subsequently, we prepared primary midgut cell cultures from last-instar larvae of *S. littoralis* as above and incubated these with the mixtures for 1 h. After washing with LPS, cells were mounted on glass slides and imaged under a confocal laser scanning microscope as mentioned above.

For each cell 5–8 z-sections were taken at 2 μm-spacing. The microvillar pole of the cells was manually selected in each picture and the average pixel intensity was measured using ImageJ. The ratio of fluorescence intensity in the microvillar pole over the background was calculated to reduce influence of potential inconsistencies between pictures. The impact of the incubation with the specific competing carbohydrate on lectin binding was analyzed using independent-samples *t*-test in SPSS Statistics 22 (IBM).

## Results

### Lectin binding to columnar cells

**Figure 1:**
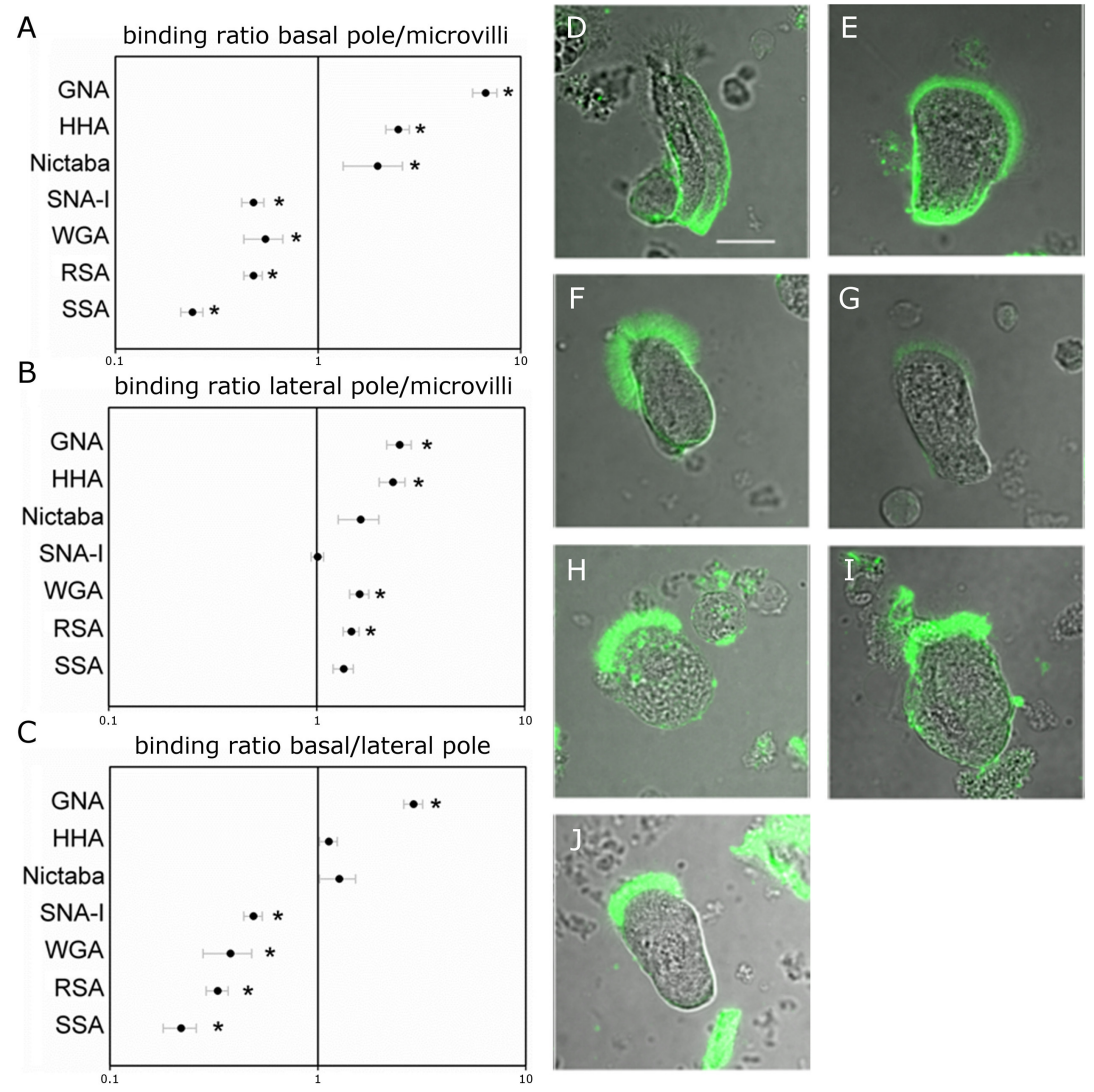
Lectin binding to different regions on the surface of the midgut columnar cells of *Spodoptera littoralis*. **(A,B)** Ratio of FITC-labeled lectin binding to basal **(A)** or lateral **(B)** zones over the binding intensities to microvilli suggest that glycans recognized by SSA, RSA, WGA, and SNA-I are more abundant at the microvillar zone while those recognized by Nictaba, HHA, and GNA are more abundant at the basal poles. Data are presented as average values ± SEM. Asterisks indicate statistically significant differences (*p* < 0.05, independent sample *t*-test). **(C)** Comparison between the basal and lateral parts of the cell membranes indicates differential binding for WGA, RSA, HHA, and GNA. Data are presented as average values ± SEM. Asterisks indicate statistically significant differences (*p* < 0.05, independent sample *t*-test). **(D–J)** Representative confocal images of midgut columnar cells incubated with FITC-labeled lectins: GNA **(D)**, HHA **(E)**, Nictaba **(F)**, SNA-I **(G)**, WGA **(H)**, RSA **(I)**, and SSA **(J)**. Pictures of the cells are oriented to show the microvillar zone at the top. Scale bar is 20 μm.

To analyze the distribution of different glycans, the columnar midgut cells were incubated for 1 h with FITC-labeled lectins specifically recognizing mannose oligomers (GNA and HHA), GalNAc/Gal (RSA and SSA), GlcNAc (WGA and Nictaba) and Neu5Ac(α-2,6)Gal/GalNAc (SNA-I). The carbohydrate-binding specificity of the selected lectins covers most of the glycan motifs that may be present in insect cells. Each fluorescently labeled lectin tested bound to the microvillar brush border zone at the apical side of the columnar cells (Figure 1). Measured fluorescence intensities in the microvillar region of the cell were significantly higher than the levels of autofluorescence (*p* < 0.05). To reveal spatial differences in the type of glycans present on the gut columnar cells, lectin binding to the basal pole and the lateral membranes was also quantified (Table 1). Subsequently, the relative lectin binding to the three zones of the cell membrane was calculated for each individual cell. Hereby the binding in the different zones was normalized to compensate for differences in membrane structure. This allowed grouping into two lectin clusters (Figure 1A). Four lectins bound relatively more to the apical brush border microvilli than to the basal pole: WGA by 1.8 fold (*p* = 0.001), and SNA-I and RSA by 2.1 fold, and SSA by 4.1 fold (all three *p* < 0.001). In turn, GNA, HHA, and Nictaba bound preferentially to the basal pole compared to the apical microvilli by 6.7 fold (*p* < 0.001), 2.5 fold (*p* < 0.001) and 2.0 fold (*p* = 0.049), respectively. Furthermore, (Figure 1B), SNA-I, RSA, SSA, and WGA showed a 2.1, 2.6, 3.0, and 4.6 fold higher (all four *p* < 0.001) binding to the microvilli compared to the lateral membranes, respectively. Only in the case of GNA, the fluorescence intensity was significantly higher (2.8 fold, *p* < 0.001) in the lateral zone compared to the apical brush border zone. Furthermore, as shown on Figure 1C, we observed a higher binding to the basal pole compared to the lateral membranes for GNA, HHA (*p* < 0.001), WGA (*p* = 0.018), and RSA (*p* = 0.027).

**Table 1:** Lectin binding intensities to different regions on the surface of columnar cells and stem cells from the larval midgut of *Spodoptera littoralis*.

	**Columnar cells**				**Stem cells**	
		** 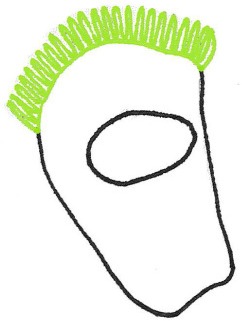 **	** 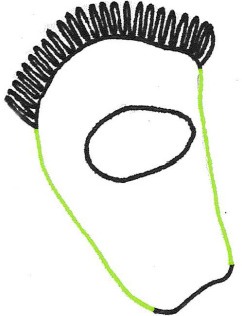 **	** 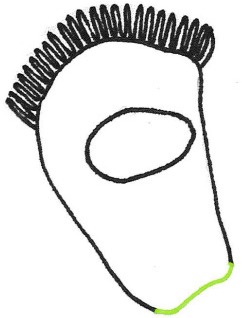 **	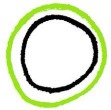	
**Lectin**	***n***	**Apical membrane**	**Lateral membrane**	**Basal membrane**	***n***	**Cell perimeter**
SNA-I	19	5.0 ± 2.7	2.0 ± 0.8	1.9 ± 0.5	10	1.5 ± 0.2
WGA	15	25.9 ± 22.7	4.1 ± 1.5	6.3 ± 3.7	6	6.8 ± 2.3
Nictaba	9	4.6 ± 1.8	5.3 ± 2.3	7.6 ± 4.8	10	11.9 ± 5.2
RSA	26	20.3 ± 9.4	6.2 ± 4.1	8.9 ± 5.4	7	20.9 ± 8.1
SSA	21	75.0 ± 25.8	14.5 ± 8.5	17.5 ± 10.7	11	14.2 ± 5.6
HHA	22	27.1 ± 10.0	28.1 ± 14.6	56.6 ± 34.1	8	20.3 ± 5.9
GNA	19	2.3 ± 0.9	6.2 ± 2.6	14.4 ± 7.5	12	11.3 ± 8.3

*Values are mean pixel intensities measured in the manually selected apical (microvillar), basal and lateral zone of the membrane of columnar cells as well as in the cell membrane (perimeter) of stem cells. Pixel intensities are average ratios of fluorescence intensities in the green-indicated regions on the surface of the midgut cells relative to background fluorescence ± standard deviations*.

**Figure 2:**
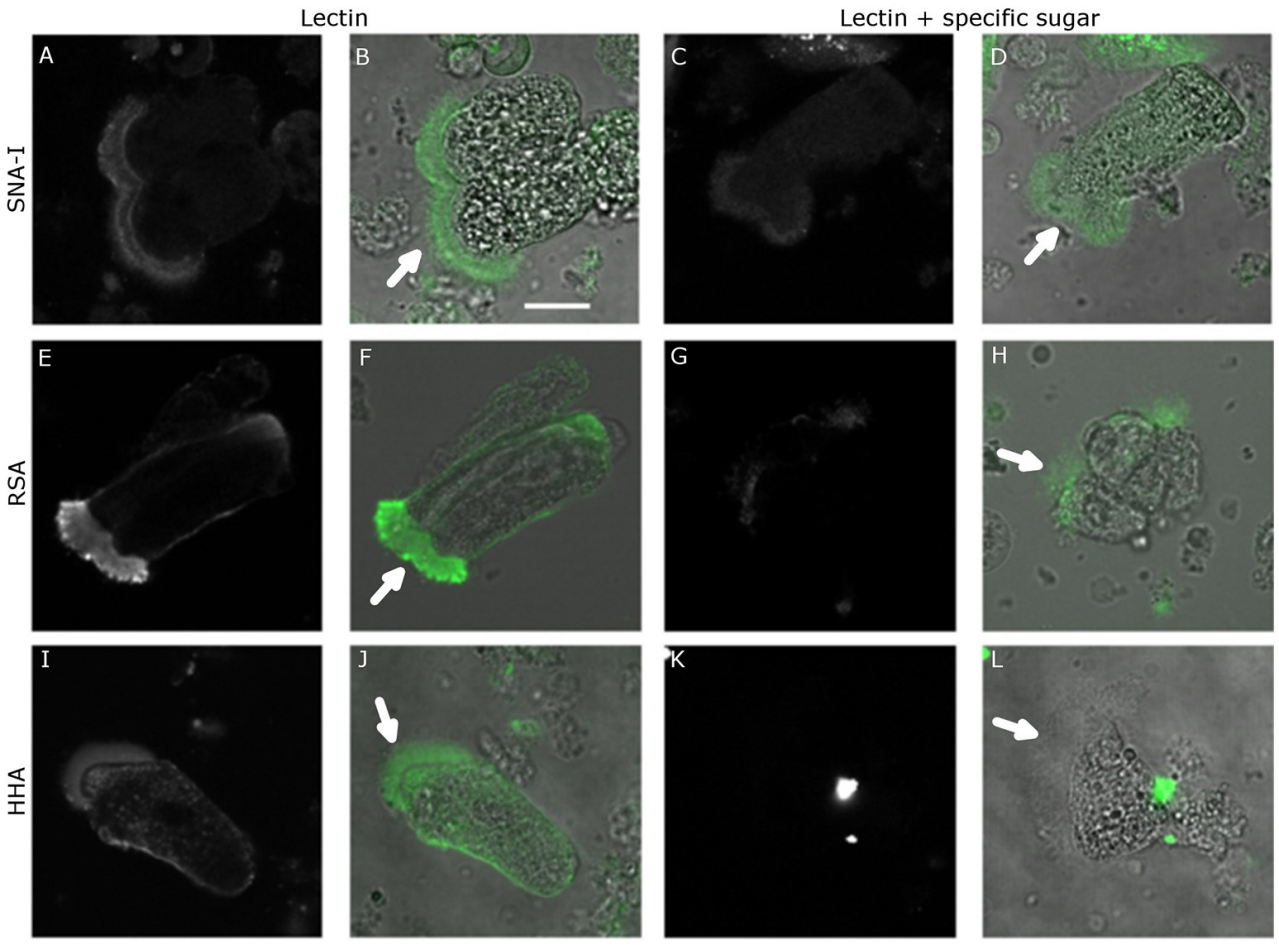
Lectin binding specificity. Confocal microscopy images of FITC-labeled lectin binding to the surface of the midgut columnar cells of *Spodoptera littoralis* after pre-incubation with competing carbohydrates. **(A–D)** Binding of SNA-I without **(A,B)** or after **(C,D)** pre-incubation with SiaLacNAc. **(E–H)** Binding of RSA without **(E,F)** or after **(G,H)** pre-incubation with GalNAc. **(I–L)** Binding of HHA without **(I,J)** or after **(K,L)** pre-incubation with yeast mannans. In each case competing carbohydrates reduced the lectin binding, indicating that the lectin binding was mediated by glycans present on the surface of the midgut cells. Scale bar is 20 μm. The arrows indicate the apical microvilli.

As shown in Figure 2, Table 2, the pre-incubation of HHA, RSA, and SNA-I with their competing carbohydrate (yeast mannans, GalNAc, and 2,6-sialyllactosamine) resulted in a significant reduction in the binding to the microvillar pole. In addition, the pre-incubation with a high concentration of a non-specific carbohydrate (100 mM GlcNAc) did not show a reduction of RSA binding to the microvilli, while the lectin interaction was significantly reduced after pre-incubation using the same concentration of the competing carbohydrate (GalNAc) (Table 2). These results indicated that lectin binding to the microvilli is glycan dependent, or at least partially glycan dependent.

**Table 2:** Inhibitory effect of carbohydrates on the binding of different lectins to the apical membrane of columnar cells from the larval midgut of *Spodoptera littoralis*.

	***n***	**Mean fluorescence ± SD**	**Binding inhibition (%)**	***p*-value**
SNA-I	6	2.85 ± 1.00	49.8 ± 9.5	0.008
SNA-I + 20 mM 6′SiaLacNAc	6	1.43 ± 0.27		
RSA	5	6.41 ± 0.88	36.9 ± 20.6	0.001
RSA + 20 mM GalNAc	3	4.05 ± 1.32		
RSA + 100 mM GlcNAc	12	8.30 ± 3.30	42.1 ± 20.0	0.002
RSA + 100 mM GalNAc	13	4.81 ± 1.66		
HHA	9	3.90 ± 1.18	62.1 ± 11.5	<0.001
HHA + 2 mg/ml yeast mannans	8	1.48 ± 0.45		

*Measurement of reduction of lectin binding to the apical membrane of columnar cells after incubation with competing carbohydrates (SiaLacNAc, GalNAc or yeast mannans) or high concentrations of a non-competing carbohydrate (GlcNAc). Values are ratios of average fluorescence intensities relative to background ± SD. Binding inhibition (%) was calculated and significance was analyzed using t-test*.

### Lectin binding to stem cells

**Figure 3:**
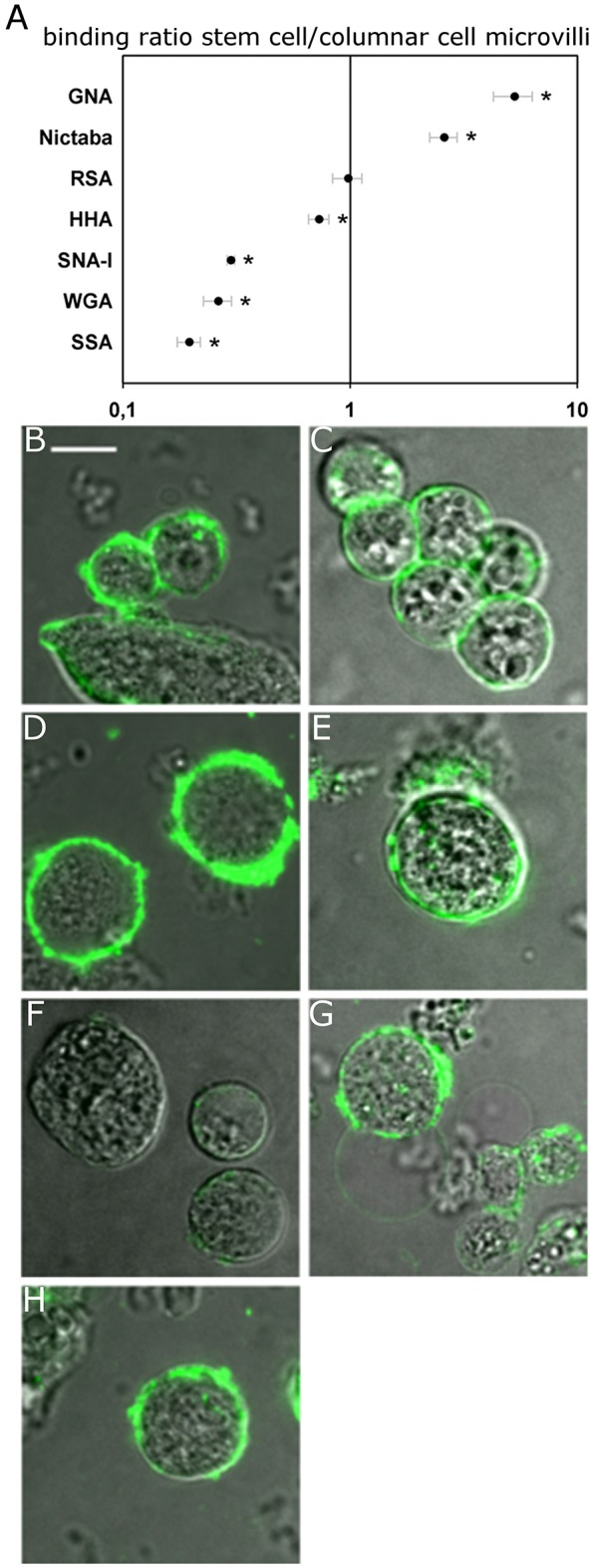
Lectin binding to the surface of the midgut stem cells of *Spodoptera littoralis*. **(A)** Ratio of fluorescence intensity in cell membranes of the stem cells over the binding intensities to microvilli of columnar cells indicates differential lectin binding to the two types of cells. Data are presented as average values ± SEM. Asterisks indicate statistically significant differences (*p* < 0.05, independent sample *t*-test). **(B–H)** Representative confocal images of stem cells incubated with FITC-labeled lectins: GNA **(B)**, Nictaba **(C)**, RSA **(D)**, HHA **(E)**, SNA-I **(F)**, WGA **(G)**, and SSA **(H)**. Scale bar is 10 μm.

In the next step, the binding of fluorescently labeled lectins to the different zones of the cell surface was compared between columnar cells and stem cells to reveal potential changes in glycan composition related with the differentiation process (Figure 3). This approach revealed that SSA, SNA-I, and WGA, and to a lesser extent HHA, bound with a relatively lower intensity to the perimeter of the stem cells compared to the apical microvillar zone of the columnar cells by 5.1, 3.8, 3.3 (all three *p* < 0.001) and 1.4 (*p* = 0.034) fold, respectively. In contrast, Nictaba and GNA bound more strongly to the perimeter of the stem cells than to the brush border microvilli of the columnar cells by 5.3 (*p* = 0.001) and 2.6 fold (*p* < 0.001), while for RSA there was no difference (*p* = 0.448).

Additionally, we observed that HHA and SNA-I bound with a higher intensity to both the basal and lateral surfaces of columnar cells, while the result was opposite for RSA and Nictaba. Compared to the perimeter of the stem cells, the normalized binding of GNA and WGA was higher in the lateral membrane zones but it was not significantly different in the basal zones (Table 1).

## Discussion

The specificity by which lectins recognize and bind certain glycan moieties makes them powerful tools to study the distribution of glycans in tissues and on cells. In this paper we used an array of seven lectins to study the spatial distribution of carbohydrate structures in different zones of the cell membrane of midgut columnar cells: mannose oligomers (GNA and HHA), GalNAc/Gal (RSA and SSA), GlcNAc (WGA and Nictaba) and Neu5Ac(α-2,6)Gal/GalNAc (SNA-I). In addition, differences in the glycan pattern between differentiated columnar cells and undifferentiated stem cells were analyzed.

The presence of mannose, GlcNAc and GalNAc in insect glycans is well-established. Mannose residues are found in the majority of the insect *N*-glycans (Aoki et al., 2007; Rendić et al., 2008; Dojima et al., 2009; Walski et al., 2016, 2017), in glycophosphatidylinositol anchors (Varki et al., 2009) and in a minor fraction of *O*-glycans (Aoki et al., 2008). GlcNAc oligomers are present in chitin or in the chitobiose core of all *N*-glycans, while terminal GlcNAc can be found in glycosphingolipids, a minor fraction of the complex and hybrid *N*-glycans as well as in *O*-glycans (Aoki et al., 2007, 2008; Varki et al., 2009). GalNAc residues have been reported in *D. melanogaster O*-glycans and a majority of glycosphingolipids (Varki et al., 2009) and on very rare complex *N*-glycans (Aoki and Tiemeyer, 2010; Kurz et al., 2015). In line with the previous findings, we observed binding of the lectins to these carbohydrate residues in the midgut cells of *S. littoralis* (Lepidoptera). Previously, the binding of several of these lectins to the midgut epithelium was shown *in vivo* or on isolated whole midguts (Fitches et al., 2001; Hamshou et al., 2010a, 2013; Caccia et al., 2012). However, due to the organization and architecture of the cells in the intact tissue of the midgut, with a peritrophic matrix in the lumen, a lamina and muscle layer at the basal side and cells placed in a layered epithelium (Hakim et al., 2010), only the apical zone of the cell is available for lectin binding. The use of primary cell cultures, containing the individual cells of the midgut as free cells suspended in the liquid medium, allows to study the distribution of glycan structures over the whole cell membrane with unhindered accessibility to all external sides of the cell.

Our observations indicated a clear distribution of lectin binding to the surface of the *S. littoralis* midgut epithelial cells, and this was apparently related to the carbohydrate specificity of lectins. The differences in membrane surface between the apical brush border, lateral and basal zones did not allow direct comparison of the measured intensities. To compensate for the differences in membrane folding, the intensities were normalized. This normalization is an estimation and not completely exact, but it gives a clear indication of the differences in lectin binding to the three zones.

Pre-incubation of SNA-I, HHA, and RSA lectins with the respective complementary glycoconjugates 2,6-sialyllactosamine, yeast mannans (mannose polysaccharides) and *N*-acetylgalactosamine, resulted in a significant reduction of the lectin binding to the microvilli. This result demonstrated that the binding of lectins to the insect midgut epithelial cells is at least partially mediated through binding to glycan moieties present on the cell surface. This is in accordance with previous experiments by Caccia et al. (2012) and Hamshou et al. (2013) where it was shown that binding of HHA to *S. littoralis* midgut cells and binding of RSA to CF-203 midgut cells was significantly reduced after pre-incubation of the lectins with their complementary glycoconjugates. Similarly, pre-incubation of SSA with GalNAc or asialomucins significantly reduced the cell toxicity of the lectin to CF-203 cells (Hamshou et al., 2010a). And studies with mutated SNA-I showed that the carbohydrate-binding sites are necessary for the insecticidal activity of the lectin (Shahidi-Noghabi et al., 2008).

The lectin binding patterns in this study suggest that GalNAc and terminal GalNAc residues (most likely on *O*-glycans and/or glycosphingolipids) are more abundant in the apical region of the columnar midgut cells, whereas mannose residues (most likely on high- and oligomannose *N*-glycans) appear to be more abundant in the basal region of these cells. Similarly, lectin binding studies in *D. melanogaster* indicated that GalNAc moieties are more abundant on the apical/luminal regions of the gut and tracheal cells (Theopold et al., 1996; Tian and Ten Hagen, 2007b). This distribution over the midgut epithelial columnar cells, reflected in the differences in the glycan motifs between the basal and lateral membranes, might be linked to the presence of different membrane proteins and by extent different functions of these membrane zones. While the basal and lateral membrane are involved in cell-cell and cell-matrix interactions, being processes in which glycans play specific roles (Varki and Lowe, 2009), the apical membrane is the primary site for many physiological, biochemical and biological interactions. The presence of clathrin and the release of digestive enzymes at the apical brush border are essential for the digestion and uptake of nutrients. In addition, interaction with toxins and entomotoxic lectins takes place at the apical membrane. The Cry1 toxin from *B. thuringiensis* (*Bt*) was show to bind two GalNAc modified membrane proteins present in the apical brush border and binding of the *Bt* toxin was dependent on their *N*-glycosylation (Jurat-Fuentes and Adang, 2004; Perera et al., 2009). Similarly, experiments with the highly insecticidal RSA lectin identified four putatively glycosylated proteins, modified with GalNAc-moieties, associated with apoptosis as potential targets (Hamshou et al., 2013). In contrast, the mannose binding GNA lectin crosses the epithelial barrier and passes into the insect hemolymph where it can bind its targets and induces systemic toxic effects (Powell et al., 1998; Caccia et al., 2012).

Glycosylation is one of the important factors regulating the targeting of proteins in cells. Some examples showed that both *N*- and *O*-glycans are essential for either apical or basal sorting, but the impact of glycosylation seems to be protein specific (Alfalah et al., 1999; Huet et al., 2003; Potter et al., 2006). For instance, *N*-glycosylation is required for apical sorting of Mouse Fc/LDL-receptor chimera in MDCK cells (Gut et al., 1998). However, for neurotrophin, *O*-glycans drive its apical sorting and their removal results in an exclusive basolateral targeting (Yeaman et al., 1997). Moreover, gut expression of *pgant5* coding for polypeptide GalNAc-transferase 5 was essential for fruit fly viability (Tran et al., 2012). Syed et al. (2012) reported that the luminal deposition of putatively *O*-glycosylated proteins is required for proper development and growth of the *D. melanogaster* hindgut. A specific distribution in the glycan profiles was also observed for vertebrate epithelial cells in which the apical membranes are enriched with glycosphingolipids (Füllekrug and Simons, 2004; Schuck and Simons, 2004).

A special observation in this study was the specific binding of SNA-I on the brush border pole of the columnar midgut cells, suggesting the occurrence of sialylated glycans. However, the presence of sialylated glycans in insects has been controversial for many years and only in a few analyses, where high enough sensitivity was achieved, the presence of sialylated *N*-glycans could be unambiguously detected (Roth et al., 1992; Aoki et al., 2008; Aoki and Tiemeyer, 2010). Functional α-2,6-sialyltransferases and Sia synthases are present in insects (Repnikova et al., 2010; Islam et al., 2013; Kajiura et al., 2015) and sialylated glycans were found but to a low amount which is most likely due to the lack of enzymes necessary for the synthesis of ManNAc (UDP-*N*-acetylglucosamine 2-epimerase), a key intermediate for Neu5Ac synthesis (Angata and Varki, 2002; Koles et al., 2009). Since insect genomes, so far available, contain all other enzymes necessary for the process of sialylation (*N*-acetylmannosamine kinase, *N*-acetylneuraminic acid phosphate synthase, CMP-*N*-acetylneuraminic acid synthase and α2,6-Sialyltransferase), it can be expected with some certainty that the presence of ManNAc or sialic acid among nutrients would enable glycan sialylation (Angata and Varki, 2002). This phenomenon of sialylation due to the presence of sialic acid in the medium was previously observed in cultured insect cell lines (Hollister et al., 2003). Likewise, it is possible that the specific binding of SNA-I may be enhanced by sialylated proteins present in the food of the caterpillars. Indeed the artificial diet used contains bovine milk κ-casein, which is a sialylated protein (Holland et al., 2005). Upon food intake, this casein protein is present in the midgut and may have enhanced the sialylation of proteins in the columnar epithelial cells in the midgut of *S. littoralis*.

With the undifferentiated stem cells, we observed an intriguing difference in the motifs of lectin binding to the surface compared to the differentiated columnar midgut cells. It was clear that SSA, HHA, WGA, and SNA-I bound relatively less to the perimeter of the undifferentiated round stem cells as compared to the brush border of the differentiated columnar cells, while the reverse situation was observed for Nictaba and GNA. No differences in lectin staining were observed in the case of RSA. Because of the striking differences in the binding patterns between lectins that share similar carbohydrate-binding specificities such as RSA and SSA, or GNA and HHA no absolute conclusions can be drawn so far with respect to the differences in glycosylation profiles between stem cells and columnar cells, and further research on this theme is intriguing. However, it should be noted that related lectins can show subtle glycan differences in their specificity: for example, GNA and HHA both react strongly with yeast mannans, but HHA strongly binds to galactomannans and linear α1,3-linked mannan, while the binding of GNA for these glycans is much lower (Kaku et al., 1990). Compared to SSA, RSA has a preference for terminal non-reducing GalNAc residues (Skamnaki et al., 2013). Nonetheless, our data were clear in the fact that a difference in polarization of the glycan distribution could be observed between these two cell types. Indeed we observed an even distribution of the fluorescence over the cell surface in stem cells, while the differentiated columnar cells showed a clear polarization of the lectin binding. Thus, it can be concluded that differentiation is associated with polarization and that this polarization is associated with differences in glycan distribution. In addition, it was interesting to note that under the same conditions as with the columnar cells, the binding of SNA-I was negligible at the perimeter of the midgut stem cells. We believe that it is of interest to further validate this observation for the potential use of this lectin [SNA-I binding Neu5Ac(α-2,6)Gal/GalNAc] as a marker for differentiated cells. Hence, our data are a first step toward a better understanding of the importance of the glycosylation patterns for the development of the insect midgut especially in related to the cell polarity and differentiation process.

## Author contributions

TW, KD, and KC performed the experimental analysis. EV and GS supervised the study and were involved in critical analysis of the data, manuscript corrections and discussions. All authors contributed to the writing of the manuscript. All authors read and approved the final manuscript.

### Conflict of interest statement

The authors declare that the research was conducted in the absence of any commercial or financial relationships that could be construed as a potential conflict of interest.
